# PP42 Implementing A Sustainable Enhanced Recovery From Surgery Pathway After Hip And Knee Arthroplasty: A Budget Impact Analysis

**DOI:** 10.1017/S0266462324002149

**Published:** 2025-01-07

**Authors:** Melanie Lloyd, Ilana Ackerman, Ian Harris, Justine Naylor, Peter Lewis, Richard de Steiger, Anthony Wan, Rachelle Buchbinder, Zanfina Ademi

## Abstract

**Introduction:**

Increasing resources are devoted to osteoarthritis surgical care in Australia annually, with significant expenditure attributed to hip and knee arthroplasties. Safe, efficient, and sustainable models of care are required. This study aimed to determine the impact on healthcare costs of implementing an enhanced short-stay model of care (ESS-MOC) for arthroplasty at a national level.

**Methods:**

Budget impact analysis was conducted for hospitals providing arthroplasty surgery over the years 2023 to 2030. Population-based sample projections obtained from clinical registry and administrative datasets of individuals receiving hip or knee arthroplasty for osteoarthritis were applied. The ESS-MOC assigned 30 percent of eligible patients to a shortened acute-ward-stay pathway and outpatient rehabilitation. The remaining 70 percent received a current practice pathway. The primary outcome was total healthcare cost savings post-implementation of the ESS-MOC, with return on investment (ROI) ratio and hospital bed-days utilized also estimated. Costs are presented in Australian dollars (AUD) and United States dollars (USD), at 2023 prices.

**Results:**

Estimated hospital cost savings for the years 2023 to 2030 from implementing the ESS-MOC were AUD641 million (USD427 million) (95% CI: AUD99 million [USD66 million] to AUD1,250 million) [USD834 million]). This corresponds to a ROI ratio of 8.88 (1.3 to 17.9) dollars returned for each dollar invested in implementing the care model. For the period 2023 to 2030, an estimated 337,000 (261,000 to 412,000) acute surgical ward bed-days, and 721,000 (471,000 to 1,028,000) rehabilitation bed-days could be saved. Total implementation costs for the ESS-MOC were estimated at AUD72 million (USD46 million) over eight years.

**Conclusions:**

Implementation of an ESS-MOC for eligible arthroplasty patients in Australia would generate significant cost and healthcare resource savings. This budget impact analysis demonstrates a best practice approach to comprehensively assessing value, at a national level, of implementing sustainable models of care in high-burden healthcare contexts. Findings are relevant to other settings where hospital stay following joint arthroplasty remains excessively long.

